# Differential Expression Analysis for Pathways

**DOI:** 10.1371/journal.pcbi.1002967

**Published:** 2013-03-14

**Authors:** Winston A. Haynes, Roger Higdon, Larissa Stanberry, Dwayne Collins, Eugene Kolker

**Affiliations:** 1 Bioinformatics & High-Throughput Analysis Laboratory, Seattle Children's Research Institute, Seattle, Washington, United States of America; 2 Data-Enabled Life Sciences Alliance International (DELSA Global), Seattle, Washington, United States of America; 3 Department of Mathematics and Computer Science, Hendrix College, Conway, Arkansas, United States of America; 4 Seattle Children's, Predictive Analytics, Seattle, Washington, United States of America; 5 Departments of Biomedical Informatics & Medical Education and Pediatrics, University of Washington School of Medicine, Seattle, Washington, United States of America; New York University, United States of America

## Abstract

Life science technologies generate a deluge of data that hold the keys to unlocking the secrets of important biological functions and disease mechanisms. We present DEAP, Differential Expression Analysis for Pathways, which capitalizes on information about biological pathways to identify important regulatory patterns from differential expression data. DEAP makes significant improvements over existing approaches by including information about pathway structure and discovering the most differentially expressed portion of the pathway. On simulated data, DEAP significantly outperformed traditional methods: with high differential expression, DEAP increased power by two orders of magnitude; with very low differential expression, DEAP doubled the power. DEAP performance was illustrated on two different gene and protein expression studies. DEAP discovered fourteen important pathways related to chronic obstructive pulmonary disease and interferon treatment that existing approaches omitted. On the interferon study, DEAP guided focus towards a four protein path within the 26 protein Notch signalling pathway.

## Author Summary

The data deluge represents a growing challenge for life sciences. Within this sea of data surely lie many secrets to understanding important biological and medical systems. To quantify important patterns in this data, we present DEAP (Differential Expression Analysis for Pathways). DEAP amalgamates information about biological pathway structure and differential expression to identify important patterns of regulation. On both simulated and biological data, we show that DEAP is able to identify key mechanisms while making significant improvements over existing methodologies. For example, on the interferon study, DEAP uniquely identified both the interferon gamma signalling pathway and the JAK STAT signalling pathway.

## Introduction

High throughput technologies, such as next generation sequencing, microarrays, mass spectrometry proteomics, and metabolomics, are capable of evaluating the expression levels of thousands of genes, proteins, or metabolites in an individual run. As a result, the life sciences are experiencing a massive influx of data, exponentially increasing the size of databases [1]–[3]. Currently, databases contain millions of data sets from transcriptomics and thousands of from proteomics [4]–[10]. Differential expression analysis, the comparison of expression across conditions, has become the primary tool for finding biomarkers, drug targets, and candidates for further research. Typically, gene expression data have been analyzed on a gene-by-gene basis, without regard for complex interactions and association mechanisms. Ignoring the underlying biological structure diminishes the power of analysis, obscuring the presence of important biological signals.

### Biological Pathways

**Figure 1:**
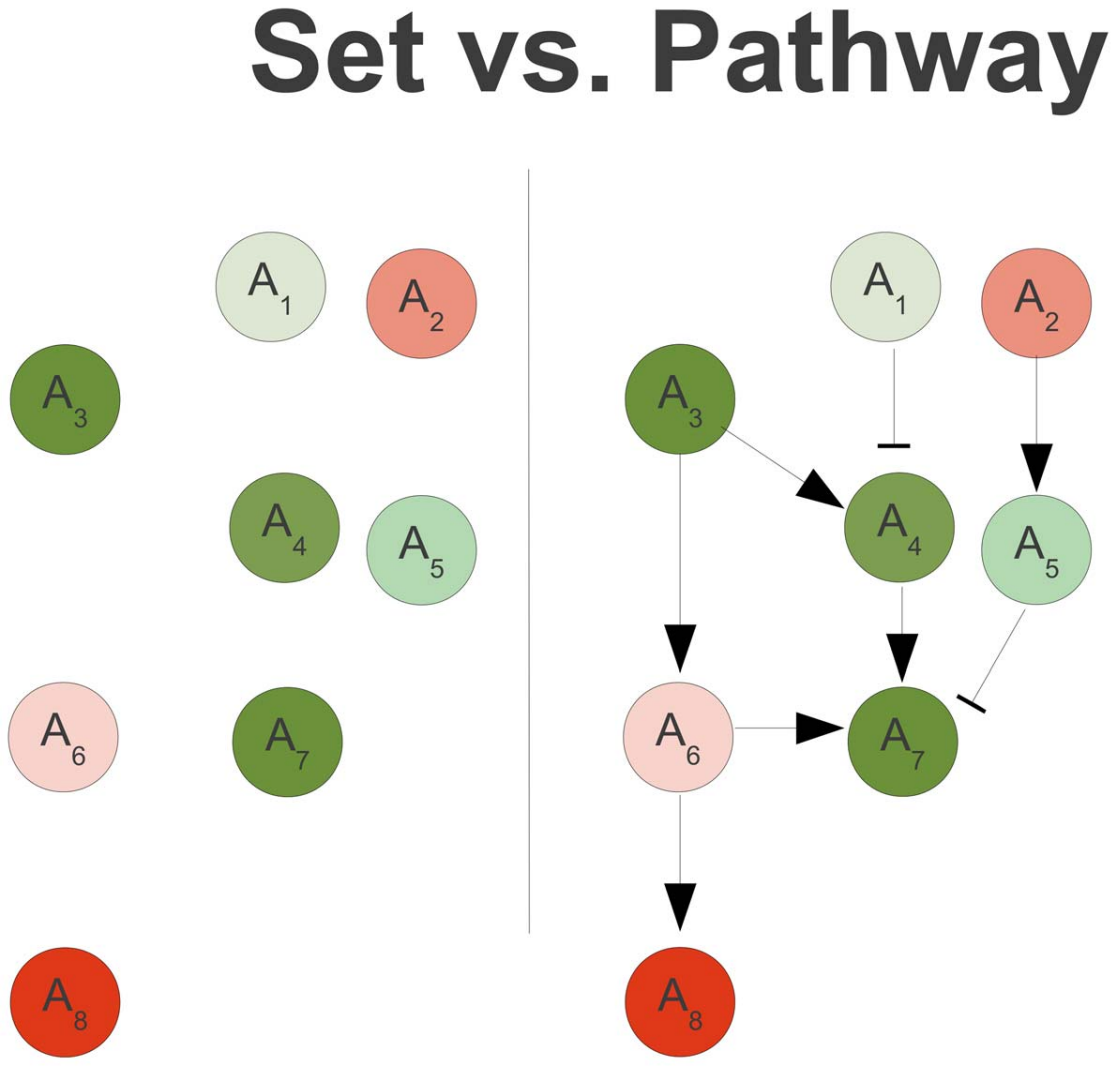
Set *vs.* pathway. Coloration from green to red represent differential expression levels, where dark green corresponds to high over expression and dark red indicates severe under expression. Edges with arrows and bars represent catalytic and inhibitory relationships, respectively. Considering *A_1_..A_8_* as one set results in inconclusive patterns of gene expression. By considering pathway relationships, *A_3_A_4_A_7_* is recognized as a path of differentially expressed genes.

Genes and proteins can be grouped into different categories on the basis of many traits: sequence, function, interactions, etc.. Grouping genes by biological pathway is often the most relevant approach to biologists. For this study, we represent biological pathways as directed graphs, where the nodes are biological compounds and the edges represent their regulatory relationships, either catalytic or inhibitory. A catalytic edge exists when expression of the parent node increases expression of the child node (i.e. *A_3_* is a parent to child *A_4_* with a catalytic edge, *Figure 1*). In an inhibitory relationship, expression of the parent node decreases expression of the child node (i.e. *A_1_* is a parent to child *A_4_* with an inhibitory edge, *Figure 1*). Further, we define a path as a connected subset of the pathway (i.e. *A_3_A_4_A_7_* is a path, *A_1_A_2_A_3_* is not, *Figure 1*). We use the term path to signify either a simple path or a simple cycle, where the term simple implies no repeated nodes.

While biological pathways have long been known, recent experimental data and computational advances have elucidated many previously uncharacterized mechanisms. Repositories contain information about thousands of biological pathways, with each pathway containing up to several hundred proteins [11]–[14]. Identifying the handful of pathways most relevant to a particular data set is an important challenge. The primary assumption of this paper is that biologically relevant pathways are characterized by co-regulated differential expression of their paths.

### Gene Set Analysis

Currently, the most popular approach to connect expression data to pathways is through gene set analysis. Gene set analysis methods consider sets of genes simultaneously as opposed to the gene-by-gene basis commonly used in differential expression analysis. One of the most prominent set-based methods is Gene Set Enrichment Analysis (GSEA), where the identified genes are ranked based on expression values [15], [16]. Significance of enriched gene sets is determined from a maximum running sum, which is calculated for each gene set by simultaneously walking down the ranked gene list and incrementing or decrementing the score on the basis of set membership. Other approaches calculate set based scores through different metrics and distributions [17]–[21]. Some of these methods compare gene sets relative to others (known as enrichment analysis or competitive approaches) while others compare individual gene sets across conditions without regard for other sets (known as self -contained approaches) [22].

The major limitation of set-based approaches in their application to pathway datasets is that they neglect the graph structure of the pathway. For example, in *Figure 1*, sporadic patterns of expression in nodes *A_1_..A_8_* would prevent identification of significant differential expression by set analysis. Considering the additional information contained in the edges, it becomes clear that *A_3_A_4_A_7_* represents a path with similar differential expression from reactants to products. Consequently, *A_3_A_4_A_7_* represents a differentially expressed path and may possess biological significance, but is unlikely to be identified as such by set based approaches.

### Pathway Analysis

We define pathway analysis as any approach which identifies patterns of differential expression in a data set by considering pathway structure. In pathway analysis, researchers are generally interested in identifying pathways associated with a biological condition and determining the components of those pathways that explain the association. Thus, hypothesis testing can be viewed as a two-step procedure: first, test an entire pathway for differential expression; second, identify the path providing the greatest contribution to that differential expression. Recent approaches to pathway analysis test the generic hypothesis of a pathway differential expression without identifying specific paths [23]–[31].

One of the most popular methods for pathway analysis, signalling pathway impact analysis (SPIA, *Table 1*) combines a set analysis score with a cumulative pathway score [23], [24]. The pathway score is calculated by summing all edges in the graph. Catalytic and inhibitory relationships are considered by using a multiplier on the expression values. While this score takes into consideration the graph structure of pathways, it includes all possible paths, rather than just differentially expressed paths. For example, in *Figure 1*, the SPIA score would be based on the combination of path scores for *A_3_A_4_A_7_*, *A_1_A_4_A_7_*, *A_2_A_5_A_7_*, *A_3_A_6_A_8_*, and *A_3_A_6_A_7_* and the set score for *A_1_..A_8_*.

**Table 1:** Term definitions.

Term	Definition
COPD	Chronic obstructive pulmonary disease
DEAP	Differential expression analysis for pathways. The approach presented in this paper.
False discovery rate	A statistical measure in multiple hypothesis testing which controls for the number of falsely rejected null hypothesis.
False positive rate	See Type I error rate.
GSEA	Gene set enrichment analysis [16]
High differential expression	In text, low differential expression refers specifically to simulations with *μ* = 1
KEGG	Kyoto Encyclopedia of Genes and Genomes [13]. A pathways database.
Low differential expression	In text, low differential expression refers specifically to simulations with *μ* = 0.25
MOPED	Model organism protein expression database [10] http://moped.proteinspire.org
*p*-value	Probability of obtaining the test statistic by random chance.
PANTHER	Protein analysis through evolutionary relationships [11]. A pathways database.
Path	A subset of a pathway which is connected by biochemical interactions.
Pathway	A series of biochemical interactions used in biological systems to perform biological functions.
Power	The frequency of occurrence of true positives. Equivalent to one minus the type II error rate (false negative rate).
Reactome	A pathways database [14]
SPIA	Signaling pathway impact analysis [24]
SPIRE	Systematic proteomics investigative research environment [40] http://www.proteinspire.org
Type I error rate	The frequency of occurrence of type I errors, false positives.
μ	Pathway effect. The average of ‘on’ genes within a pathway.

Protein interaction permutation analysis, designed for siRNA experiments, calculates the significance of the number of interactions in a network for which both genes are “hits” [25]. Recently, *Zhao et al.* introduced an approach that includes pathway structure in the analysis of genome wide association studies [26]. However, neither of these methods are directly applicable to expression data. Other pathway analysis approaches calculate set enrichment scores, but weight gene products based on their correlation with neighboring genes in the pathway [27], [28]. Alternatively, other approaches integrate omics data over pathways, but encode all expression data as −1, 0, or 1, limiting the information utilized from experimentation [29]. A mixed linear model presents an advanced approach to the hypothesis test, but is limited to acyclic models and implementation remains complex [30]. Like SPIA, all of these approaches account for the pathway structure as a whole, rather than identifying differentially expressed paths. To our knowledge, popular commercial pathway tools (i.e. Ingenuity Pathway Analysis, BioBase, GeneGo, Metacore, Ariadne) currently offer no methods that directly incorporate pathway analysis.

### Significance Testing

High-throughput data analysis typically falls into the category of *p*>>*n* problems, where the number of genes or proteins, *p*, is considerably larger than the number of samples, *n*. Pathway and gene set analysis methods have the added complexity that gene expression within pathways is often highly correlated. Therefore, the statistical analysis approaches described above typically rely on random permutations of biological replicates in order to preserve expression correlation structure. However, the small sample size limits the number of possible permutations and, hence, the precision of *p*-value estimates. In addition, permutation tests are only applicable to simple experimental designs. Utilizing a random rotation approach circumvents these issues [32]–[34].

### Proposed Solution: DEAP

In this study, we present a new pathway analysis method, Differential Expression Analysis for Pathways (DEAP). The primary assumption of DEAP is that patterns of differential expression in paths within a pathway are biologically meaningful. DEAP calculates the path within each pathway with the maximum absolute running sum score where catalytic/inhibitory edges are taken as positive/negative summands. To assess the statistical significance, we use a random rotation. Similar to other pathway analysis methods, DEAP tests a generic hypothesis of overall pathway differential expression. Contrary to current methods, DEAP identifies the most differentially expressed path to provide a refined focus for further biological exploration.

## Results

### DEAP Algorithm

**Figure 2:**
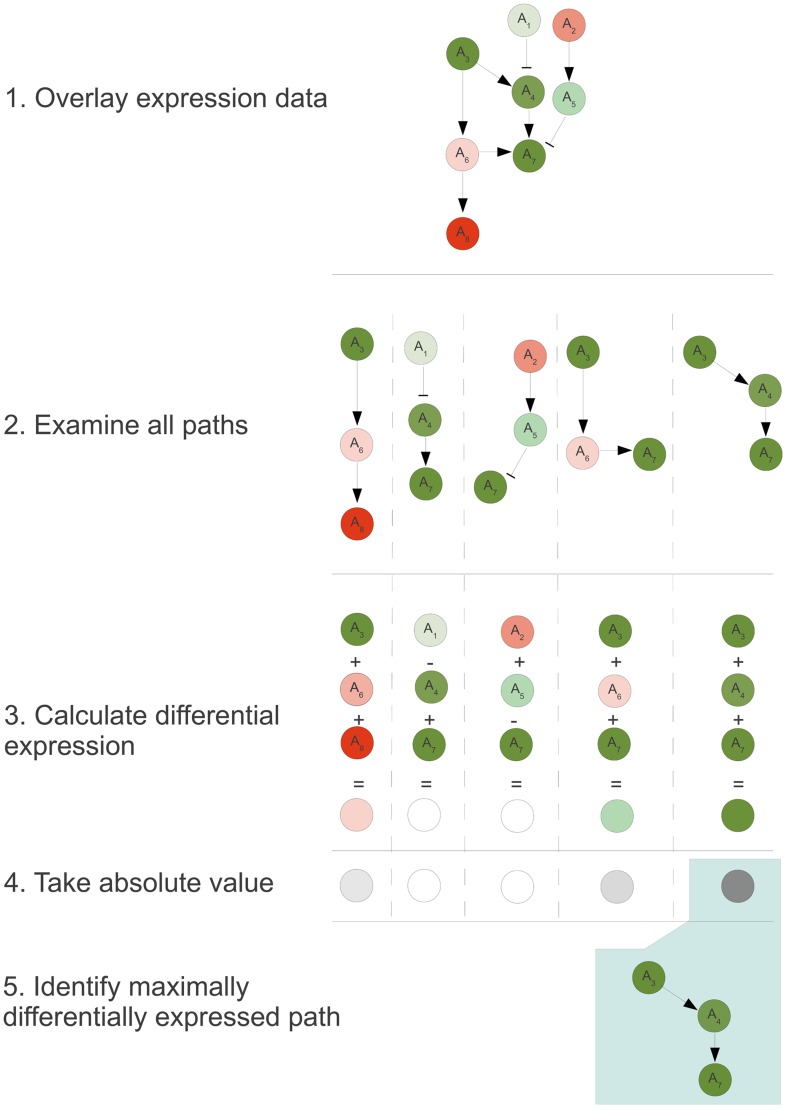
DEAP algorithm workflow. A visual representation of the DEAP algorithm workflow described in *Methods*.

As illustrated in *Figure 2*, the DEAP algorithm begins by overlaying expression data onto the pathway graph (*Figure 2.1*). Every possible path from the graph is independently examined (*Figure 2.2*). A recursive function calculates the differential expression for each path by adding or subtracting all downstream nodes with catalytic or inhibitory relationships, respectively (*Figure 2.3*).

**Figure 3:**
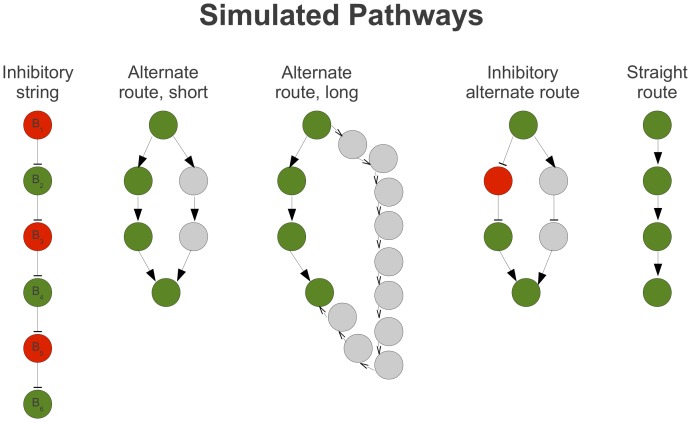
Simulated pathways. Five pathways designed to test analysis approaches are illustrated. Nodes labelled in green and red were built around distributions with *μ* of +*X* and −*X*, respectively, where *X* represents a numerical value. Gray nodes represent data sampled from the standard normal distribution. Edges with arrows and bars represent catalytic and inhibitory relationships, respectively.

As an example, the score for the path containing all nodes in the inhibitory string in *Figure 3* *(left)*, where green = +1 and red = −1, is calculated as:
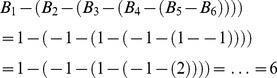
(1)The absolute value of the expression level is utilized as the DEAP score (*Figure 2.4*) to determine the path with maximal differential expression (*Figure 2.5*). The DEAP algorithm returns both the maximum absolute value and the path associated with that maximum value. The algorithm is formalized in *Methods* *: DEAP Algorithm*.

DEAP scores for different pathways are not directly comparable due to size and structure differences among pathways. Thus, we employ a self-contained approach which individually assesses the significance of each pathway. Generating a null distribution is complicated by the low number of samples relative to gene identifications and the correlation of gene expression within pathways. Most existing approaches use permutation tests to preserve the correlation between genes; however, small sample size limits their effectiveness. We use random rotation to circumvent these issues [32]–[34]. Our random rotation implementation is applicable to a wide range of complex experimental designs with multiple conditions and replicates. The significance levels are adjusted for multiple comparisons using the false discovery rate method of Storey and Tibshirani [35].

For each pathway in the analysis, DEAP outputs its score, the corresponding *p*-value, and the path with the maximum absolute score (see examples in *Files S1, S2*). The open source implementation (licensed under the GNU Lesser General Public License v3.0) of this algorithm is available in Supplemental Materials (*File S3*).

### DEAP Validation 1: Simulated Data on Simulated Pathways

Data from the five pathways illustrated in *Figure 3* were simulated as described in *Methods*. Algorithmic performance was measured in terms of power, the percentage of times each differentially expressed pathway was identified as significant (*p*<0.05), which is equivalent to one minus the type II error rate. The power of DEAP was compared to GSEA and SPIA, the two most popular gene set and pathway analysis methods, respectively. Comparative analysis of these methods included four key parameters: the overall effect (mean of ‘on’ genes, *μ*), variation in individual gene effects (*σ^2^_g_*), sample size (*n*), and type I error rate.

**Figure 4:**
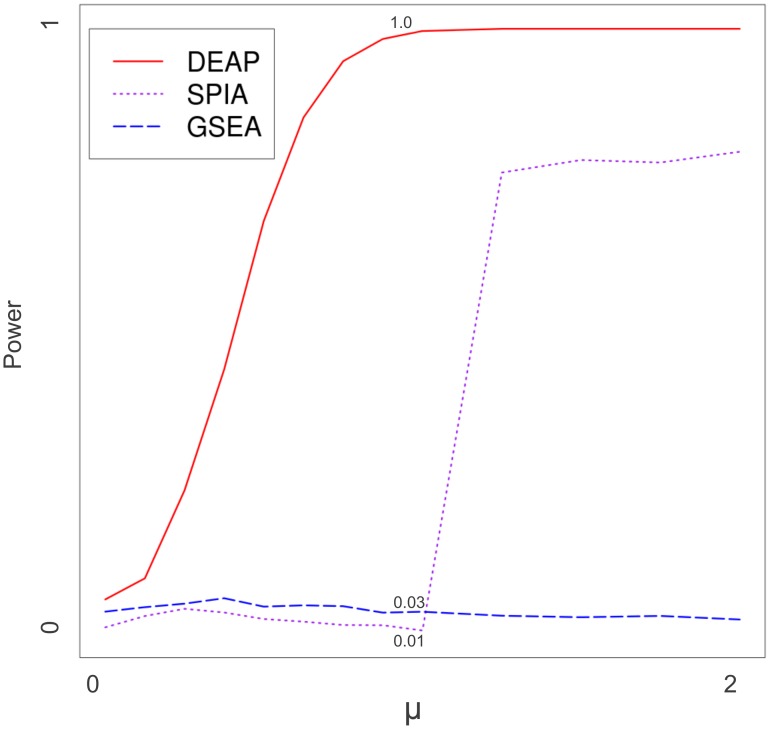
Power curve, variable pathway effect. Performance of GSEA, SPIA, and DEAP are compared as pathway effect (μ) changes. Specific values are indicated at *μ* = 1. Power (y-axis) is the ratio of simulations, out of 5000 (5 pathways, 1000 simulations each), which were identified as significant (*p*<0.05). Constants were *σ^2^_g_* = 0 and sample size = 10.

Regardless of the level of differential expression, DEAP was consistently more powerful than were other approaches (*Figure 4*). For small *μ* values (low differential expression), the power of DEAP was approximately twice that of GSEA and SPIA, demonstrating improved sensitivity. For *μ* = 1 (high differential expression), DEAP had an increase in power over both GSEA and SPIA of two orders of magnitude. At *μ* = 1.25, the performance of SPIA improved substantially, approaching that of DEAP on all pathways except the long alternate route where SPIA was confounded by noise (*Figure S1*). Across the board, GSEA performed poorly because GSEA did not consider pathway structure and is dependent on comparisons to other pathways.

**Figure 5:**
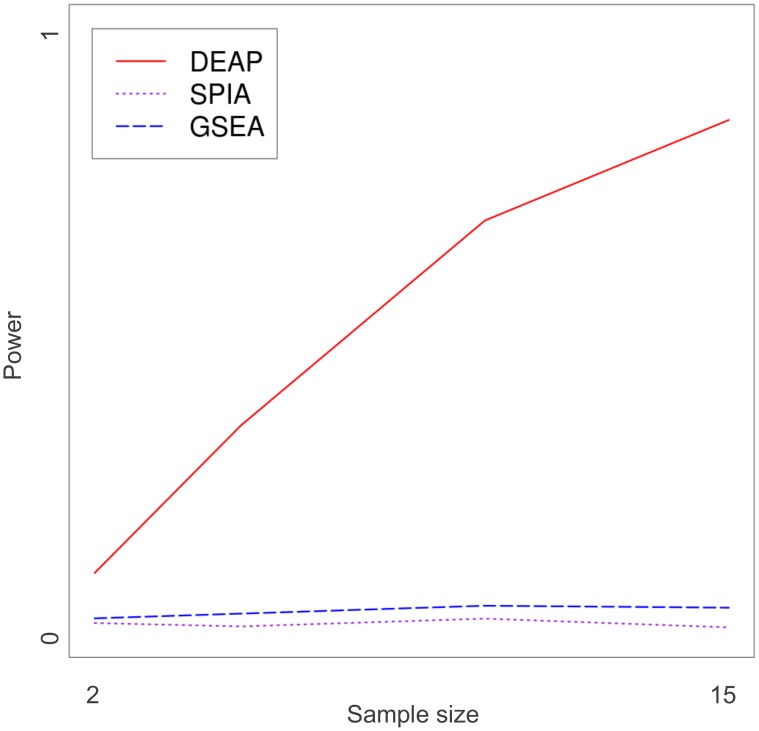
Power curve, variable gene variance. Performance of GSEA, SPIA, and DEAP are compared as gene variance (*σ^2^_g_*) changes. Power (*y*-axis) is the ratio of simulations, out of 5000 (5 pathways, 1000 simulations each), which were identified as significant (*p*<0.05). Constants were *μ* = 0.5 and sample size = 10.

**Figure 6:**
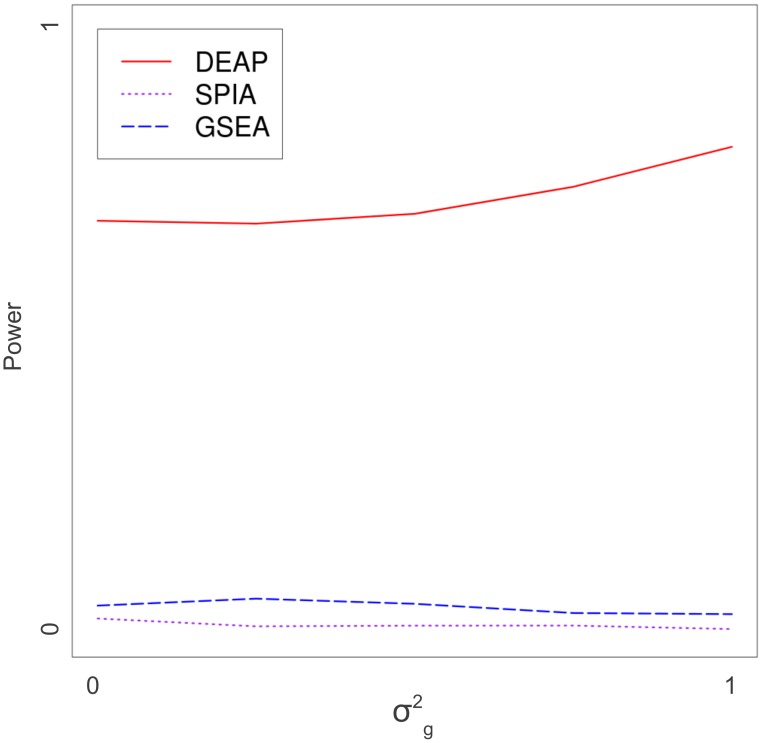
Power curve, variable sample size. Performance of GSEA, SPIA, and DEAP are compared as sample size changes. Power (*y*-axis) is the ratio of simulations, out of 5000 (5 pathways, 1000 simulations each), which were identified as significant (*p*<0.05). Constants were *σ^2^_g_* = 0 and *μ* = 0.5.

Sample size and within-gene variance also have significant effects on the performance of the algorithms. As sample size (*n*) grew, the power of DEAP relative to other approaches increased, particularly in pathways containing inhibitory edges (*Figure 5*). As variance (*σ^2^_g_*) increased, DEAP exhibited minor increases in power (*Figure 6*). Further, DEAP consistently outperformed GSEA and SPIA as variance increased.

**Figure 7:**
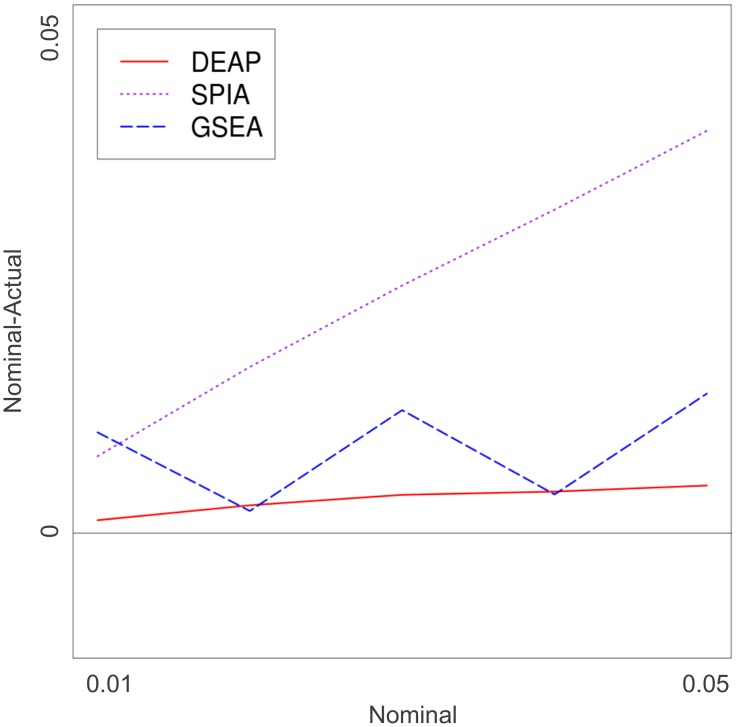
Type I error.

To estimate the type I error rate, we simulated random data under the null hypothesis (*μ* = 0, *σ^2^_g_* = 0, *n* = 10). The plots in *Figure 7* displays type I error rates with respect to the nominal values. SPIA was notably more conservative for every pathway structure. The performance of both GSEA and DEAP was on target; however, DEAP was more conservative on pathways with inhibitory edges (*Figure S4*).

An additional advantage of DEAP is the ability to identify the maximally differentially expressed path of the pathway. For the simulated data with *μ* = 1 and *μ* = 2, DEAP identified the entire differentially expressed path 99% and 100% of the time, respectively. For example, the long alternate route contains 14 proteins, but DEAP identified the differentially expressed region that contains only four, substantially reducing the search space.

In addition to comparing DEAP to GSEA and SPIA, we compared DEAP to several modifications of the DEAP algorithm, which were altered as follows: scores normalized by pathway length; all weights set to +1; and sum taken across the entire pathway. We also compared DEAP to a set-based implementation with rotation. DEAP had substantially higher power than all four approaches (*Table S1* and *Figures S1, S2, S3, S4*).

### DEAP Validation 2: Simulated Data on Biological Pathways

While simulated pathways provide easily controllable examples to validate DEAP as an appropriate test of the hypothesis, biological pathways bring increased complexity from which the signal must be detected. To validate DEAP on more realistic pathway structures, we simulated activity on biological pathways from the KEGG and Reactome databases [13], [14].

**Figure 8:**
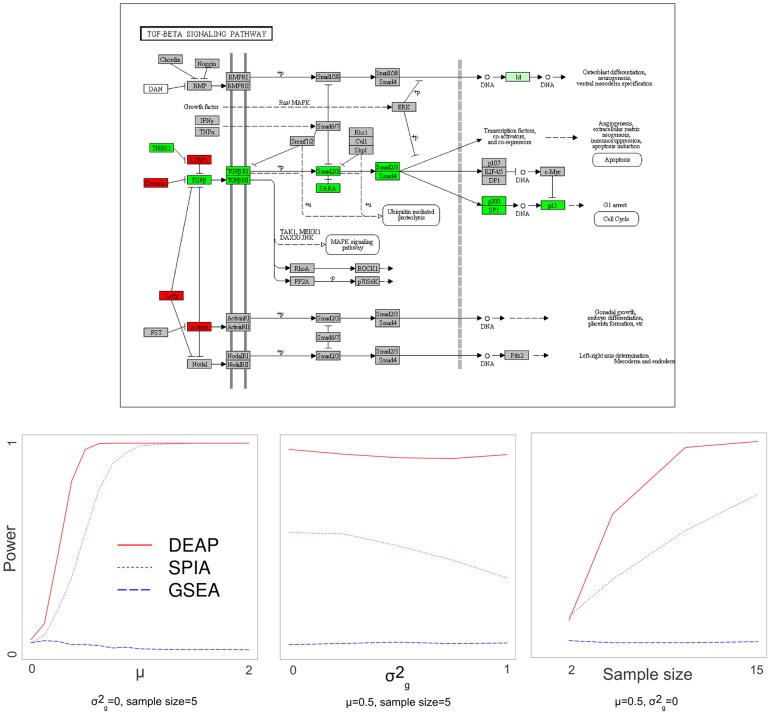
Simulated data on the TGFβ signalling pathway, power *vs.* pathway effect, variance, and sample size. At the top, the KEGG TGFβ signaling pathway is illustrated, with green, red, and grey nodes representing nodes whose simulated values were +μ, −μ, and 0, respectively [13]. The nodes are colored to indicate activity leading to G1 arrest in the cell cycle. At the bottom, power for detecting significant differential expression in this pathway is illustrated with respect to pathway effect, variance, and sample size. Figure adapted from http://www.genome.jp/kegg-bin/show_pathway?map04350 with permission from KEGG.

In the case of KEGG [13], we simulated data on the TGF-ß signaling pathway to indicate activity in the TGF- ß receptors leading to cell cycle arrest (*Figure 8*). In terms of sensitivity to the pathway effect (*μ*), variance (*σ^2^_g_*), and sample size, DEAP outperformed both GSEA and SPIA on the TGF-ß signaling pathway (*Figure 8*). Notably, increased variance diminishes the power of SPIA, but does not affect DEAP, reflecting its ability to identify signal in the noisy environments common in biological experimentation.

**Figure 9:**
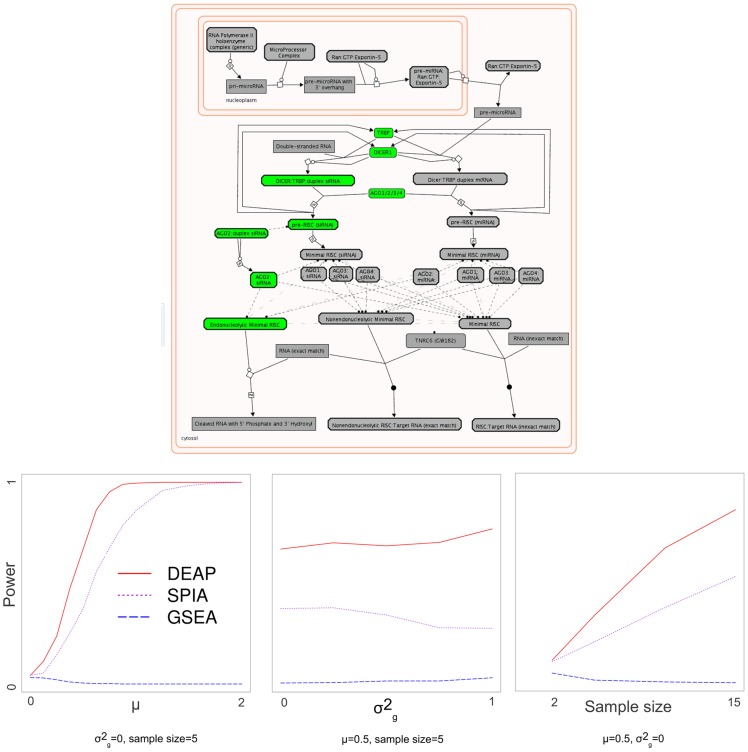
Simulated data on the post-transcriptional silencing by small RNAs, power *vs.* pathway effect, variance, and sample size. At the top, the Reactome post-transcriptional silencing by small RNAs pathway is illustrated, with green, red, and grey nodes representing nodes whose simulated values were +μ, −μ, and 0, respectively [14]. The nodes are colored to indicate activity leading to silencing by cleaved RNA with 5′ phosphate and 3′ hydroxyl. At the bottom, power for detecting significant differential expression in this pathway is illustrated with respect to pathway effect, variance, and sample size.

In the case of Reactome [14], we simulated data on the post-transcriptional silencing by small RNAs pathway from to indicate RNA cleavage (*Figure 9*). DEAP had superior performance over GSEA and SPIA in terms of all tested variables: pathway effect (*μ*), variance (*σ^2^_g_*), and sample size (*Figure 9*).

In both sets of simulated data on real biological pathways, the type I error estimate was conservative for DEAP, GSEA, and SPIA (*Figure S5*). In addition to DEAP, GSEA, and SPIA, we applied the four alternative formulations of DEAP to both sets of biological pathways and noted the consistently strong performance of DEAP (*Figures S6, S7*).

### DEAP Validation 3: Biological Data on Biological Pathways

To verify that the simulated data effects are biologically relevant, we also applied DEAP to two sets of biological data on biological pathways. The experimental data are from a transcriptomic study of interferon [36], [37] and a proteomic study of chronic obstructive pulmonary disease (COPD). We applied DEAP, GSEA, and SPIA to identify differentially expressed pathways from the PANTHER database [11]. Pathway associations with the phenotypes were determined based on a literature review using Google Scholar (details in *Methods* *: Biological Data Validation*).

We analyzed a microarray expression data of cells of radio-insensitive tumors that had been treated with interferon [36], [37]. DEAP identified six pathways with known literature associations with interferon while GSEA identified five and SPIA identified none (*Table 2*). The two most clearly relevant pathways for this transcriptomics data set were interferon gamma signalling, as the cells had been stimulated with interferon; and JAK STAT signalling, the pathway being studied by the authors of the microarray study [36], [37]. Unlike GSEA and SPIA, DEAP identified these pathways as significantly differentially expressed. The lack of overlap between the pathways identified by GSEA and DEAP is indicative of the different hypotheses being tested by these two approaches, with GSEA focusing on non-specific differential expression among pathway genes and DEAP focusing on differential expression among pathway connected genes. As such, these two approaches should be viewed as complementary approaches that can be simultaneously utilized to augment biological discovery.

**Table 2:** Results from Interferon microarray data analysis using GSEA, SPIA, and DEAP [36], [37].

Pathway	GSEA	SPIA	DEAP
*Interferon gamma signaling*			S
*JAK STAT signaling* [47]			S
*PDGF signalling* [48]			S
*Notch signalling* [38]			S
*Interleukin signalling* [49]			S
*General transcription regulation* [50]			S
Beta1 adrenergic receptor signalling			S
*Histamine H1 receptor mediated signalling* [51]	S		
*Oxytocin receptor mediated signalling* [52]	S		
*Thyrotropin releasing hormone receptor signalling* [53]	S		
*Integrin signalling* [54]	S		
*Arginine biosynthesis* [55]	S		
Parkinson disease	S		

An *S* indicates pathway differential expression significance of *p*< = 0.05. Italic text indicates previously discovered associations between the pathway and interferon. Non-italic text indicates no known associations between the pathway and interferon. Specific p-values are found in Table S5.

**Figure 10:**
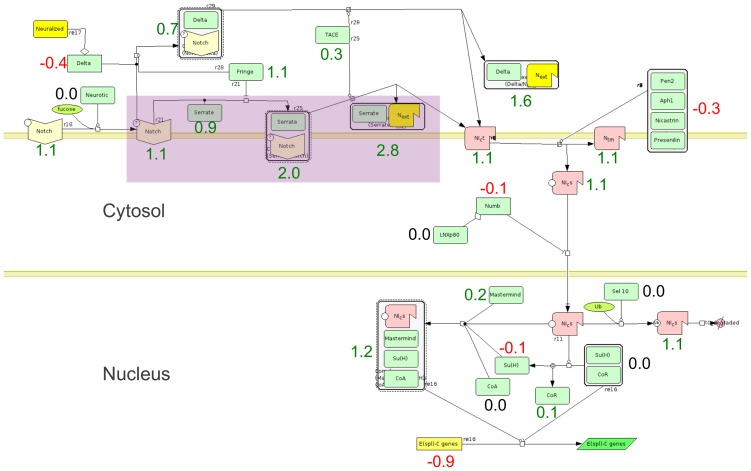
Maximally differentially expressed path identification. Maximally differentially expressed path identification by DEAP on the Notch signalling pathway. Pathway image is from PANTHER [11], [46]. The path shaded in purple was identified by DEAP as the most differentially expressed. Numerical values are log-expression ratios from the Interferon microarray study [36], [37].

Additionally, DEAP analysis of the interferon transcriptomics data uses path identification to reduce the search space for future experimentation. Consider the Notch signalling pathway, which contains 26 proteins and is known to be activated by interferon treatment [38]. GSEA and SPIA both did not identify Notch signalling as significantly differentially expressed due to generally sporadic expression patterns. However, DEAP analysis focused on consistent differential expression of 4 connected nodes and labelled Notch signalling as significantly differentially expressed (*Figure 10*). Without identifying the maximally differentially expressed path, the Notch signalling pathway would have been overlooked. Further, future experimentation can now focus on those four proteins exhibiting the most significant differential expression.

In order to illustrate DEAP on a different data type, we also analyzed a proteomics study which compared healthy smokers with patients diagnosed with COPD (*Methods* *: Biological data*, *Table 3*). On this data set, GSEA identified nine pathways, four of which had apparent associations with COPD. SPIA identified only one pathway with significant differential expression. DEAP identified 12 pathways and eight had literature-verified implications with COPD. Of notable clinical relevance to COPD is the inflammation mediated by chemokine and cytokine signalling pathway, which was identified only by DEAP [39].

**Table 3:** Results from the COPD proteomics data analysis using GSEA, SPIA, and DEAP (*Methods* *: Biological data*).

Pathway	GSEA	SPIA	DEAP
*Integrin signalling* [56]		S	S
*Interleukin signalling* [57]			S
*Inflammation mediated by chemokine and cytokine signalling* [39]			M
*Heme biosynthesis* [58]	M		M
*Ras pathway* [59]			M
*Plasminogen activating cascade* [60]			M
*De novo purine biosynthesis* [61]			M
*Ubiquitin proteasome* [62]			M
Heterotrimeric G protein signalling, rod outer segment			M
PDGF signalling			M
De novo pyrimidine ribonucleotide biosynthesis*			M
De novo pyrimidine deoxyribonucleotide biosynthesis*			M
*Muscarinic acetylcholine receptor signalling* [63]	S		
*Nicotonic acetylcholone receptor signalling* [64]	S		
*Blood coagulation* [65]	M		
DNA replication	S		
Circadian clock system	S		
Asparagine and aspartate biosynthesis	M		
Salvage pyrimidine ribonucleotides*	M		
Arginine biosynthesis*	M		

An *S* and *M* indicate pathway differential expression significance of *p*< = 0.05 and marginal significance of *p*< = 0.1, respectively. Italic text indicates previously discovered associations between the pathway and COPD. Non-italic text indicates no known associations between the pathway and COPD.

* Arginine and pyrimidine are used therapeutically to treat COPD. Specific p-values are found in Table S6.

## Discussion

DEAP takes into account the graph structure of a pathway and determines the maximally expressed path. Pathway-centric analysis by DEAP is complementary to set-based analysis of other functional categories, as seen in both biological examples (*Tables 2* *–* *3*). Application of the random rotation approach allows for accurate assessment of statistical significance of the DEAP scores. On simulated data for simulated pathways, DEAP both increased power over existing approaches and accurately controlled the false positive rate. With high differential expression, this translated to a two-fold increase in the power of DEAP over GSEA and SPIA. On simulated data applied to real biological pathways, DEAP showed the strongest performance for all levels of pathway effect, variance, and sample size. Analysis of experimental transcriptomic and proteomic data indicates that DEAP identified important pathways related to a particular disease or condition where other approaches failed, specifically identifying six pathways related to interferon and eight related to COPD. Further, DEAP uniquely identified the most expressed path of the pathway with 100% accuracy in simulated data.

Though we demonstrated DEAP on transcriptomics and proteomics studies, DEAP is widely applicable to other omics research areas (metabolomics, lipidomics, etc.) and expression technologies (next generation sequencing, RNAseq, etc.). This broad applicability extends from the flexible design of DEAP: the only required inputs are expression levels of biomolecules and corresponding pathways. Appropriate scaling of the expression levels is defined by the user. For instance, RNAseq data is very similar to spectral count proteomics data in that they are both count-based. Thus, RNAseq read counts can be used as input for DEAP in the same manner as peptide spectral counts. Further, RNA transcripts can be used in place of proteins.

To identify the most important pathways for further study, pathways can be ranked based on DEAP score significance. Specifically, future studies can be focused on the most differentially expressed paths within the pathways with the lowest false discovery rate, which can be especially beneficial when studying pathways that contain hundreds of biological compounds. Currently, DEAP is being integrated with our proteomics analysis pipeline SPIRE (http://proteinspire.org) and expression database MOPED (http://moped.proteinspire.org) [10], [40] (*Table 1*). Application of DEAP to existing and future studies has the potential to discover meaningful biological patterns.

## Methods

### Simulated Data

Expression data (presumably on a log scale) for each gene in a pathway was simulated using a multivariate normal distribution defined in *Equation 2*:
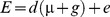
(2)In this equation d is the indicator of whether a gene is ‘on’ or ‘off’. The value of *d* is 0 if the gene is ‘off’ and +1 if the gene is up-regulated and ‘on’ and −1 if the gene is down-regulated and ‘on’. The value of *d* is determined by the predefined pathways. The variable *μ* is the mean of the absolute value of expression for ‘on’ genes and, therefore, represents the ‘pathway effect’. The value of *μ* is held constant for each gene in the pathway and across replicate samples. The variable *g* is assumed to come from a normal distribution with mean 0 and variance *σ^2^_g_*. The variance *σ^2^_g_* measures how much individual gene expression deviates from the overall ‘pathway effect’, *μ*.

The value of *g* is randomly generated (although in many of the simulations is set to 0) for each gene in the pathway, but the same value is used for replicate samples. The variable *e* is assumed to come from a normal distribution with mean 0 and variance 1 and represent random variation in gene expression. The value of *e* is randomly generated for each combination of gene and sample. The simulations varied the values *μ*, *σ^2^_g_*, and the sample size (number of independent samples of pathway data). R scripts were used to generate the simulated data [41].

Five diverse pathways were specifically created to test the efficacy of identification by different scoring methods (*Figure 3*). Gray colored nodes had unaltered values from a standard normal distribution. Nodes labelled as green and red were sampled with *μ* values of +X and −X, respectively, where X was a positive number. Simulated data and pathways are available on Dryad: doi:10.5061/dryad.qh1pg.

### Biological Data

Microarray data from a study of cells treated with interferon were acquired from the Gene Expression Omnibus (GDS3126) [6]. The sample was taken from radio-resistant tumors following treatment with a mixture of interferons [36], [37]. It was hypothesized that interferon and biochemically-related pathways would be stimulated in this data set. The expression value was the logarithm of the case/control ratio. Though microarrays measure mRNA expression, the pathways represent information in terms of proteins. Therefore, the gene identifiers in the microarray data were mapped to UniProt protein identifiers using the UniProt website [42]. Handling the one-to-many relationship of genes and proteins is discussed below (see *Methods* *: DEAP*). When duplicate probes existed for the same gene, the expression value utilized for the gene was the arithmetic mean of these probes.

The COPD proteomics data can be found at PeptideAtlas (raw data) [7] and MOPED (processed data) [10] (moped.proteinspire.org). We analyzed data from CD4 and CD8 T-lymphocytes. The control patients were healthy smokers, with an average FEV1/FVC of 82.5%. Case patients had been medically diagnosed with COPD and had an average FEV1/FVC of 42.0%. A total of 10 cases and 10 controls were utilized in this analysis. Additional experimental details can be found associated with the PeptideAtlas accession numbers in *Table S3*. On MOPED, data is stored under the experimental name “steffan_copd.” The tandem mass spectrometry data were analyzed through SPIRE with the parameters in *Table S4* [40]. Protein expression was measured by the number of peptide spectral matches identified for each protein normalized by the total number of spectra in the sample. For pathway analysis, we used the difference between the log normalized expression values.

Pathway data were downloaded from the PANTHER database [11]. A total of 165 pathways downloaded in SBML format from PANTHER pathway version 3.01. PANTHER pathways contain information about proteins, biochemicals, and other substrates. For the purposes of data interpretation, the pathways were broken into their protein components using an internally developed python script where connections of proteins through biochemical substrates were maintained as protein-protein interactions PANTHER's internal identifiers were mapped to UniProt identifiers. Ultimately, parsing of the PANTHER pathway database resulted in a graph structure in which each node represented a set of proteins that act as a set of reactants and/or products. Inhibitory or catalytic edges between two sets of proteins were determined as detailed in PANTHER.

### Rotation Sampling

We used random rotation approach to estimate the null distribution of the test statistics and compute the p-values [32]. Rotation testing has been used recently in gene set analysis as an alternative to permutation and parametric tests [33], [34]. Rotation tests have an advantage over permutation tests in that they produce reasonable results for small sample sizes and complex experimental designs. Rotation testing assumes that pathway and set data come from independent random samples of a multivariate normal distribution with mean zero under the null hypothesis. A rotation test is carried out by multiplying the original data by a random rotation matrix, calculating the test statistic, and repeating the procedure to generate a null distribution. Adjustments for an overall mean, covariates, or blocking factors are handled by performing the rotations of an orthogonal projection of the original data on to the residual space from a linear model and then transforming the rotated data back. A random rotation matrix was generated by first generating a matrix *X* of standard normal random variables and then taking the rotation matrix to be the orthogonal matrix *Q* from the *QR* decomposition of *X*. Scripts to carry out rotation testing were written using the R programming language and are available in *File S3*, released under the GNU Lesser General Public License v3.0. The user is able to input a custom design matrix which accounts for complex experimental designs with multiple conditions and replicates.

### DEAP Algorithm

Given: a current edge, all other edges in graph, expression values for all proteins:

For single channel (unpaired) data, define *E*(*x*) to be the difference between the logarithm of the arithmetic mean of expression values associated with protein *x* in the two conditions.

For two channel (paired) data, define *E*(*x*) to be the arithmetic mean of the log expression ratio(s) associated with protein *x*.

The recursive function operates as follows:

1. Recursively examine all edges in the pathway set whose reactant node is the current edge's product node.
  - If there are no such edges, set *max_recursive_* and *min_recursive_* as 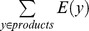 where 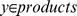 refers to each protein, *y*, contained in the edge's products.
  - Otherwise, define *max_recursive_* and *min_recursive_* as the maximum and minimum scores, respectively, returned by the recursive function.
2. Assign *max_score_* and *min_score_* as the maximum and minimum, respectively of: 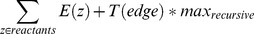 and 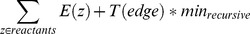
where *T*(*edge*) is the multiplier associated with the edge type (−1 or 1 for inhibition or catalysis, respectively) and 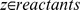 refers to each protein, *z*, contained in the edge's reactants.
3. Return the maximum of {*max_score_*, 0} and the minimum of {*min_score_*, 0}.

### DEAP Statistics

In DEAP, the maximum order (by absolute value) path is used to test the null hypothesis about the expression of the entire pathway. This claim, that the expression of one path answers questions about the expression of the pathway, is justified on two levels.

On a biological level, significant fluctuations in activity do not require differential expression of an entire pathway. For example, in *Figure 1*, *A_3_A_4_A_7_* represents a path with similar expression levels that proceeds all the way from reactants to products, a pattern that seems to be significant.

From a logical perspective, consider a pathway, *P*, as the union of all paths of the pathway, *P_1_*, *P_2_*, …, *P_K_*. Each path is completely defined by its set of edges. Note that the *k*-paths are not entirely disjoint in the sense that some paths might share the nodes and the edges. However, we require each path to have a distinct set of edges. To test the hypothesis of a differentially expressed pathway requires testing whether any of the constituent paths is differentially expressed. This corresponds to testing the family of *k*-null hypothesis. To control the family wise error rate, we use a maximum order statistic, since the probability of making at least one incorrect decision under the null is equivalent to the probability of the maximum order statistic exceeding the threshold.

To approximate a null distribution of the test statistic, s*, we performed *n* rotations of the data. For each rotation sample, we recompute the DEAP score, *s_i_*. The *p*-value is calculated as a proportion of scores that are at least as extreme as the observed score, the proportion of simulated DEAP scores whose value are greater than or equal to the observed DEAP score:
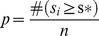



### DEAP Implementation

The DEAP algorithm was implemented to allow for efficient computation.

By maintaining global maximum and minimum values and updating their values as the recursive function proceeds, it is not necessary to examine all paths of the graph independently. Rather, we can initialize DEAP score calculations only at leaf edges, which have no upstream edges pointing to any proteins in their reactant set. To ensure that closed cycles are not missed, we track the edges which have been visited and examine additional edges until the difference of the complete edge set and the already visited edge set is empty. This greatly reduces the number of calculations per graph.

Once the recursive function has returned a maximum and minimum score for a particular edge, that score will remain constant regardless of the preceding edge except in the case of cycles (see paragraph below). Therefore, we use a dictionary mapping edges to maximum and minimum scores to prevent duplicative score calculations. After this implementation, score calculations that took several hours on particularly complex pathway structures completed in seconds.

In the case of cycles, scores may be dependent on the node of the cycle which is examined first. For these cycles, our current implementation represents a heuristic estimator rather than the exact optimal solution. Bidirectional edges are subject to this same limitation as they are equivalent to a two node cycle. Implementations that determined the exact optimal solution were prohibitively slow for practical application. Except in edge cases, the heuristic implementation will provide approximations of sufficient quality to identify significant patterns of differential expression.

Every DEAP score calculation is independent of other DEAP score calculations, so we set up processing for multi-threading. For example, on a 64-bit Intel Core i7-2720QM CPU with 8GB RAM, speed improvements of approximately 4-fold were noted for the score calculation process. Specific running time is highly dependent on expression data set size, experimental design, pathway complexity, and number of rotation testing iterations. Running DEAP on 90 simulated data files each with 10 samples, 1000 proteins, 1000 pathways, and performing 100 data rotations took 72 minutes when multi-threaded and 260 minutes when performed on a single thread.

The function tracks edges that have already been examined in a particular recursive cycle to prevent entrance into infinite loops in cyclical pathways. To control for duplicate protein identifiers, summations over the products and reactants were performed on the set of unique expression values rather than for every identifier. For example, if protein A and protein B both had expression levels of 1.743 and were both in the same protein set, then it was assumed they were the result of data duplication and 1.743 was only added to the score once. This duplication elimination was implemented primarily due to issues arising from redundant protein identifiers and potential mRNA translation into multiple proteins. For instance, the five UniProt identifiers for variants of Histone H3 (Q6NXT2, P68431, Q16695, Q71DI3, and P84243) are included in the same PANTHER pathway unit and share near identical protein sequences, so their proteomic and transcriptomic identification will be duplicated.

The algorithm was implemented in Python and is available in *File S3*, released under the GNU Lesser General Public License v3.0.

### Biological Data Validation

Accuracy of pathway associations with experimental conditions were validated using a Google Scholar literature search. The literature search was performed by searching Google Scholar (http://scholar.google.com) for a combination of the pathway name and details of the experimental condition. We continued searching Google Scholar until satisfied that the association was confirmed or felt reasonably certain that there was not yet a literature confirmed association. Once a literature association was confirmed, the most pertinent reference was retained and cited in this manuscript.

### Assumptions

The DEAP approach is based on the following fundamental assumptions:

1. The user provides expression values using an appropriately scaled metric that represent meaningful information. DEAP is independent from the calculation of individual gene expression values, with the stipulation that data be numeric, where positive values represent over-expression and negative values represent under-expression. For example, microarray expression data have been shown to be scale free in nature, so a logarithm scaled expression ratio was input to the DEAP algorithm.
2. Existing pathway knowledge is sufficient to make meaningful statements. Though pathways currently contain only a fraction of all proteins, this approach makes no attempt to expand that coverage [43].

### Existing Approaches

The GSEAlm package for the R Project, available through BioConductor, was utilized to perform GSEA analysis [44]. Pathways were transformed into a gene set matrix and multi-sample expression data were loaded appropriately. Since GSEA performs test for up- and down-regulation independently, the minimum of these two values was taken and multiplied by two to adjust for a two-tail test.

SPIA analysis was performed using the SPIA package for the R Project, available through BioConductor [45]. To convert the pathways into the SPIA format, inhibitory and catalytic relationships were formatted into the inhibition and activation matrices, respectively. Since the SPIA implementation only allowed input of single expression ratios, the arithmetic mean of expression values for each protein was input into SPIA.

## Supporting Information

Figure S1 Power vs. pathway effect for all 7 approaches for simulated data on simulated pathways.
(TIFF)

Figure S2 Power vs. sample variance for all 7 approaches for simulated data on simulated pathways.
(TIFF)

Figure S3 Power vs. sample size for all 7 approaches for simulated data on simulated pathways.
(TIFF)

Figure S4 Type I error for all 7 approaches for simulated data on simulated pathways.
(TIFF)

Figure S5 Type I error for simulated data on biological pathways.
(TIFF)

Figure S6 Power vs. pathway effect, sample size, and variance for all 7 approaches for simulated data on the KEGG TGFβ signalling pathway. Figure adapted from http://www.genome.jp/kegg-bin/show_pathway?map04350 with permission from KEGG
(TIFF)

Figure S7 Power vs. pathway effect, sample size, and variance for all 7 approaches for simulated data on the Reactome post-transcriptional silencing by small RNAs pathway.
(TIFF)

File S1 DEAP results on CF data.
(TXT)

File S2 DEAP results on COPD data.
(TXT)

File S3 Archive file of DEAP source code licensed under the GNU Lesser General Public License v3.0.
(ZIP)

Table S1 Provides a summary of approaches to pathway analysis, rationale for their inclusion, and summary of the results for simulated data.
(DOC)

Table S2 Decision justifications.
(DOC)

Table S3 PeptideAtlas accession numbers for COPD study.
(DOC)

Table S4 COPD search parameters using SPIRE.
(DOC)

Table S5 p-values for *Table 2* *: Results from Interferon microarray data analysis using GSEA, SPIA, and DEAP*.
(XLS)

Table S6 p-values for *Table 3* *: Results from the COPD proteomics data analysis using GSEA, SPIA, and DEAP*.
(XLS)
