# Correction: Differential Expression Analysis for Pathways

**DOI:** 10.1371/annotation/58cf4d21-f9b0-4292-94dd-3177f393a284

**Published:** 2013-04-26

**Authors:** Winston A. Haynes, Roger Higdon, Larissa Stanberry, Dwayne Collins, Eugene Kolker

A minor correction has been made to the function “meanExpDict”, where the conditional statement was flipped during code refactoring. Further, the instructions have been made more clear. Please view the correct File S3 here: 

You can also access the source code repository directly via this link: https://sourceforge.net/projects/deapathways/ 

