# Supplementary figures and images for: Differential Expression Analysis for Pathways
Source: PLoS Comput Biol. 2013 Mar 14;9(3):e1002967. doi: 10.1371/journal.pcbi.1002967 (PMC3597535; doi:10.1371/journal.pcbi.1002967)

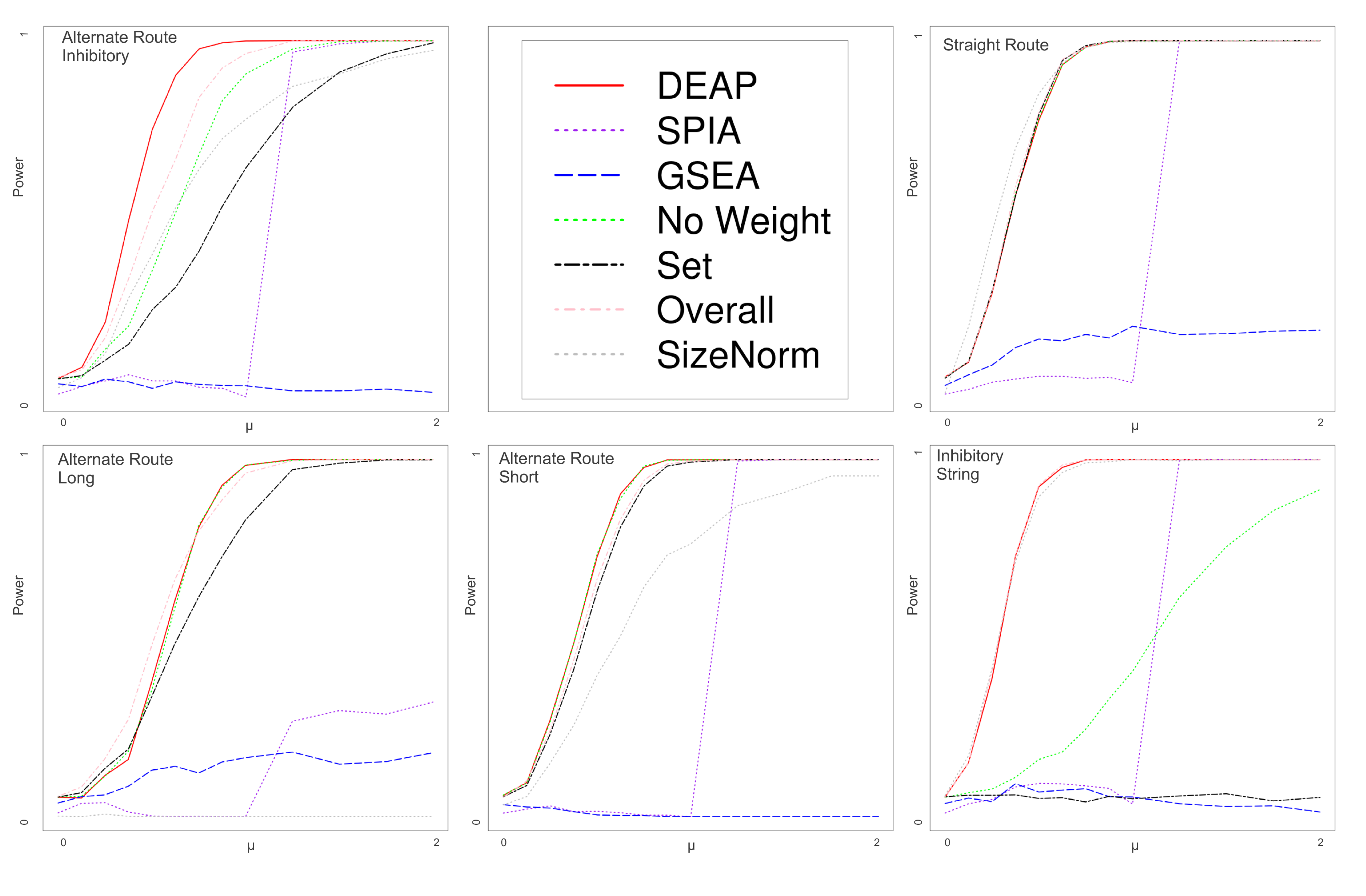

Supplement: Figure S1 — Power vs. pathway effect for all 7 approaches for simulated data on simulated pathways. (TIFF) [file pcbi.1002967.s001.tif]

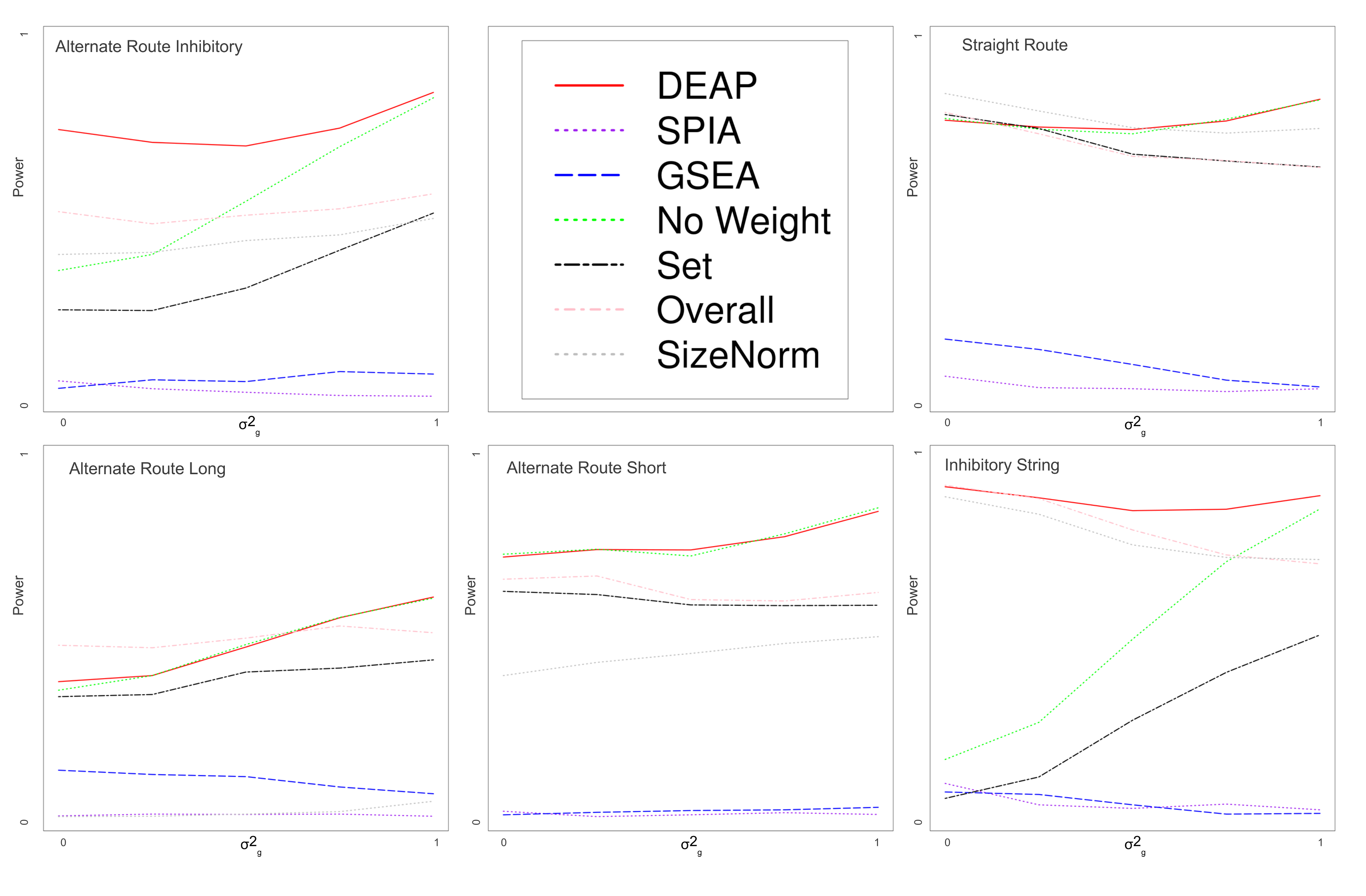

Supplement: Figure S2 — Power vs. sample variance for all 7 approaches for simulated data on simulated pathways. (TIFF) [file pcbi.1002967.s002.tif]

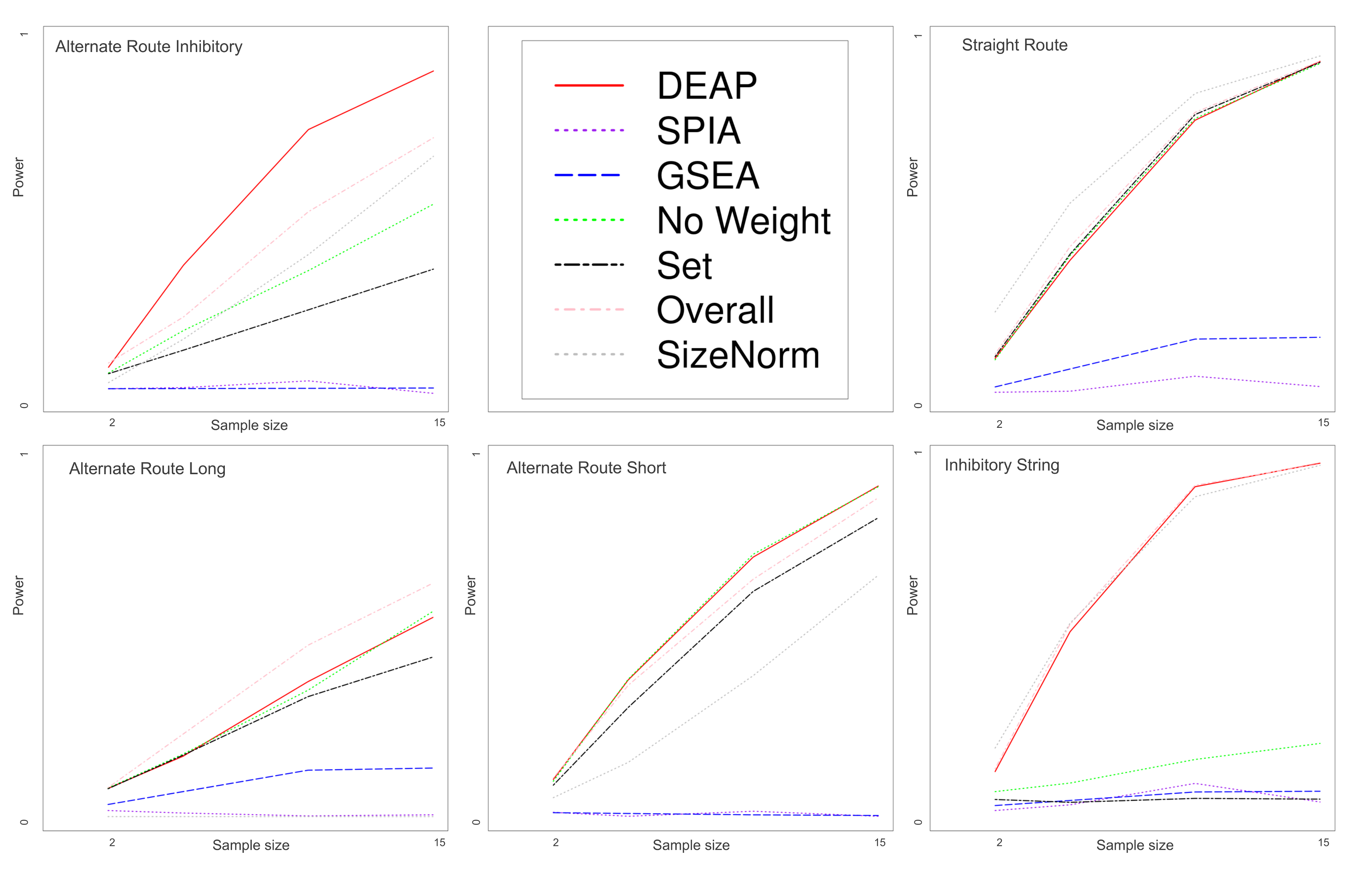

Supplement: Figure S3 — Power vs. sample size for all 7 approaches for simulated data on simulated pathways. (TIFF) [file pcbi.1002967.s003.tif]

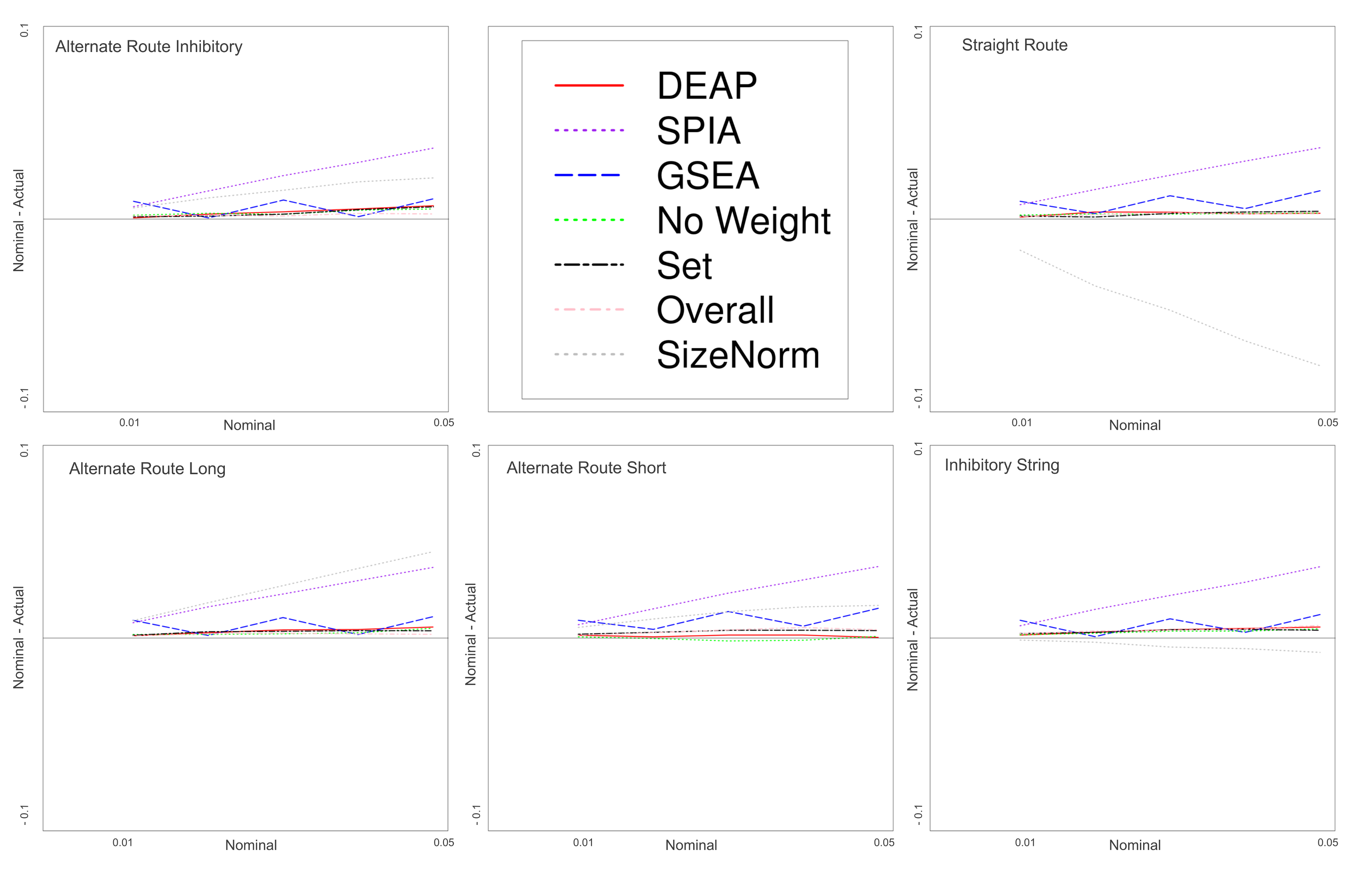

Supplement: Figure S4 — Type I error for all 7 approaches for simulated data on simulated pathways. (TIFF) [file pcbi.1002967.s004.tif]

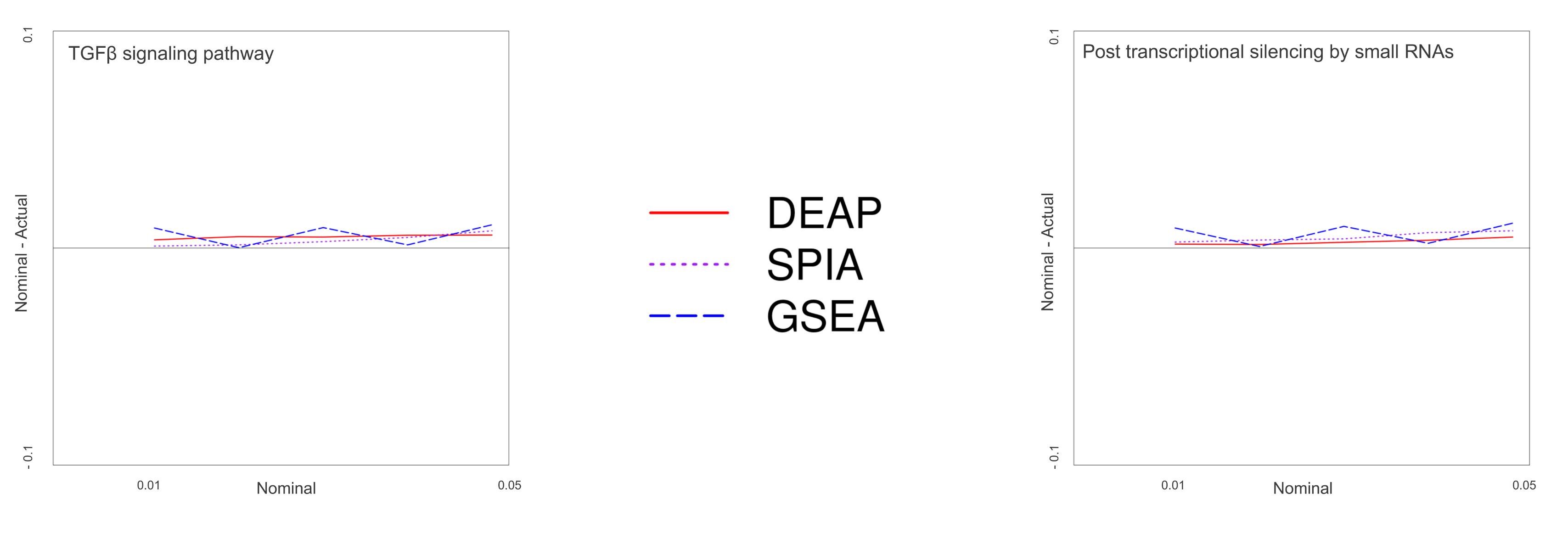

Supplement: Figure S5 — Type I error for simulated data on biological pathways. (TIFF) [file pcbi.1002967.s005.tif]

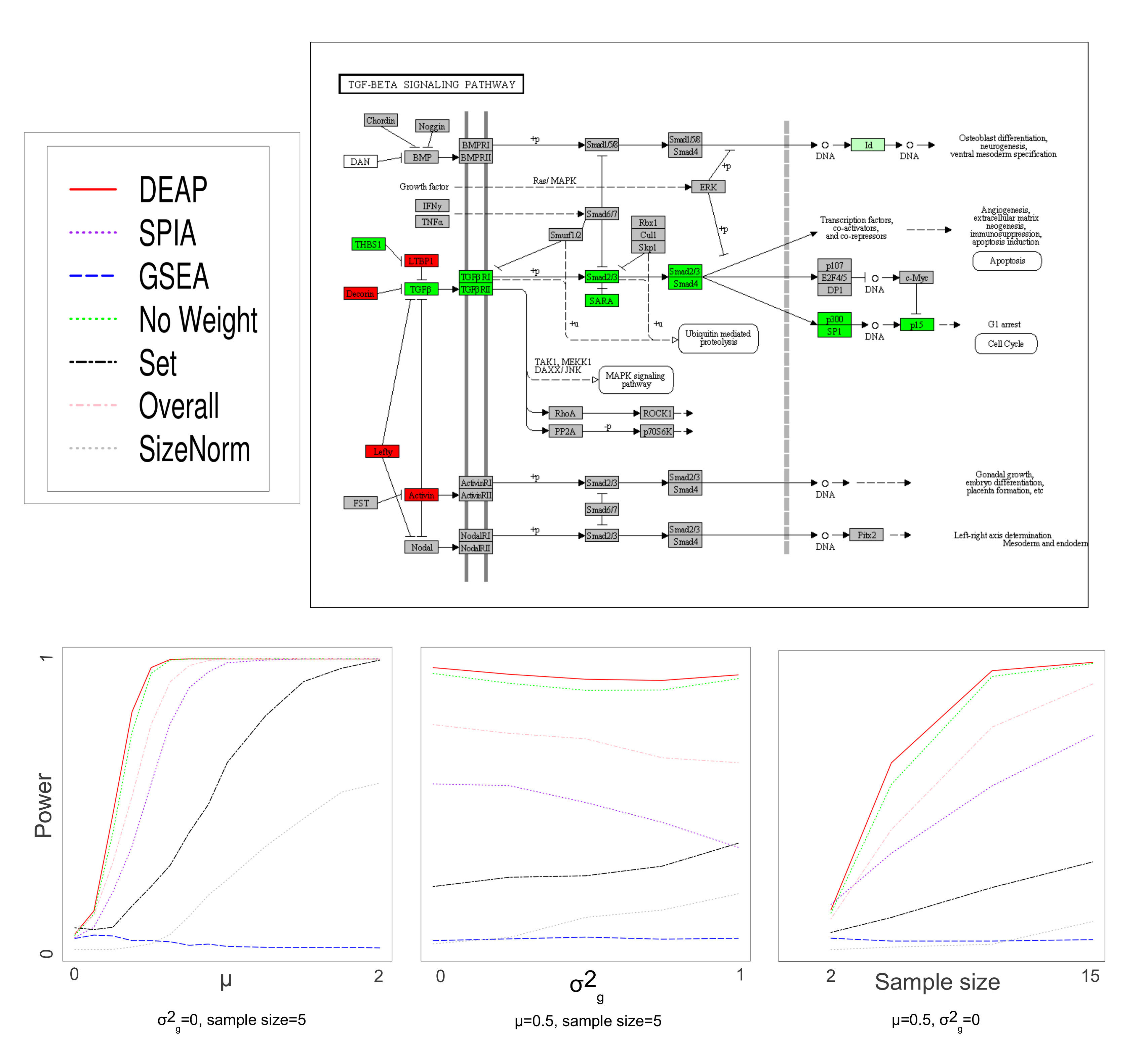

Supplement: Figure S6 — Power vs. pathway effect, sample size, and variance for all 7 approaches for simulated data on the KEGG TGFβ signalling pathway. Figure adapted from http://www.genome.jp/kegg-bin/show_pathway?map04350 with permission from KEGG (TIFF) [file pcbi.1002967.s006.tif]

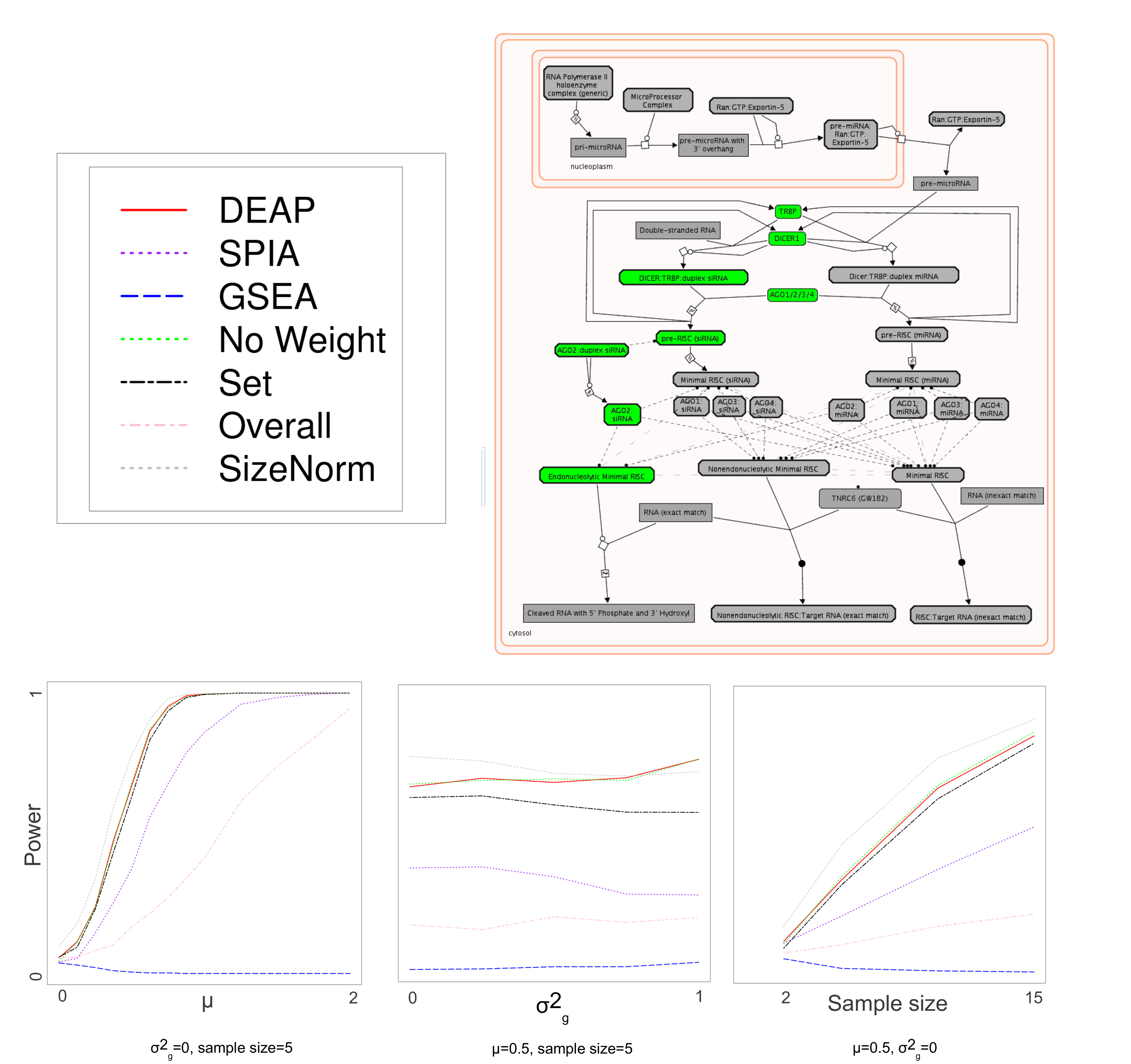

Supplement: Figure S7 — Power vs. pathway effect, sample size, and variance for all 7 approaches for simulated data on the Reactome post-transcriptional silencing by small RNAs pathway. (TIFF) [file pcbi.1002967.s007.tif]
