# Supplementary material for: Differential Expression Analysis for Pathways
Source: PLoS Comput Biol. 2013 Mar 14;9(3):e1002967. doi: 10.1371/journal.pcbi.1002967 (PMC3597535; doi:10.1371/journal.pcbi.1002967)
Supplement: Table S1 — Provides a summary of approaches to pathway analysis, rationale for their inclusion, and summary of the results for simulated data. (DOC) [file pcbi.1002967.s011.doc]

| **Name** | **Approach** | **Rationale** | **Simulated results** |
| --- | --- | --- | --- |
| Set | Sum all expression values in pathway. | Generic set analysis. | Better than GSEA on all pathways (likely due to rotation testing and non-enrichment methods).  Worse than DEAP on all pathways with inhibitory edges and alternate routes. |
| Size Norm | Normalized scores by number of proteins in pathway. P-values calculated from all pathways. | Alternative normalization. | Worse than DEAP on all pathways with alternate routes. i.e., on the long alternate route the 4 differentially expressed nodes were divided by the length of the entire, 14 node pathway. |
| No Weight | Set all edge multipliers to +1. | Without considering inhibitory edges. | Identical to DEAP on all pathways without inhibitory relationships.  Lost a significant amount of power to DEAP on pathways with inhibitory edges. |
| Overall | Running sum across entire graph. | Without considering individual paths. | Performed worse than DEAP on pathways with alternate routes. Power was diminished by including nodes without differential expression. |
