# Supplementary material for: Differential Expression Analysis for Pathways
Source: PLoS Comput Biol. 2013 Mar 14;9(3):e1002967. doi: 10.1371/journal.pcbi.1002967 (PMC3597535; doi:10.1371/journal.pcbi.1002967)
Supplement: Table S2 — Decision justifications. (DOC) [file pcbi.1002967.s012.doc]

| **Decision** | **Justification** |
| --- | --- |
| μ=1 | Point at which DEAP gained maximal power over GSEA and SPIA. |
| μ=1.25 | Point at which SPIA showed a significant increase in power. |
| μ=2 | Point at which DEAP identifies the maximally differentially expressed path with 100% accuracy. |
| *p*<=0.05 | *p*<=0.05 is a common threshold for claiming statistical significance. |
| *p*<=0.15 | *P*<=0.15 includes pathways of marginal significance for the sparse proteomics data set. |
| Why GSEA? | GSEA is a widely utilized tool for analysis of gene sets. |
| Why SPIA? | SPIA is a popular tool for analysis of pathways. Of all existing approaches, SPIA is the most similar to DEAP. |
