# Supplementary material for: Differential Expression Analysis for Pathways
Source: PLoS Comput Biol. 2013 Mar 14;9(3):e1002967. doi: 10.1371/journal.pcbi.1002967 (PMC3597535; doi:10.1371/journal.pcbi.1002967)
Supplement: Table S3 — PeptideAtlas accession numbers for COPD study. (DOC) [file pcbi.1002967.s013.doc]

| **Data set** | **PeptideAtlas acession numbers** |
| --- | --- |
| COPD- CD4 | PAe000796, PAe000821, PAe000835, PAe000874, PAe000882 |
| COPD- CD8 | PAe000838, PAe000841, PAe000867, PAe000870, PAe000875, PAe000879 |
| Healthy Smoker- CD8 | PAe000807, PAe000818, PAe000823, PAe000847, PAe000878 |
| Healthy Smoker- CD4 | PAe000805, PAe000831, PAe000840, PAe000849, PAe000858 |
