# Supplementary material for: Differential Expression Analysis for Pathways
Source: PLoS Comput Biol. 2013 Mar 14;9(3):e1002967. doi: 10.1371/journal.pcbi.1002967 (PMC3597535; doi:10.1371/journal.pcbi.1002967)
Supplement: Table S4 — COPD search parameters using SPIRE. (DOC) [file pcbi.1002967.s014.doc]

| **Variable** | **Value** |
| --- | --- |
| Cleavage | Trypsin cleavage after proline |
| Static modification | 57.02@C |
| Variable modification | 16.0@M |
| Mass error | +- 3 Da |
| Fasta file | Human UniProt, downloaded 6-27-2011 |
